# Rising STARS: Developing Youth Engagement in Crystallography

**DOI:** 10.1063/4.0000977

**Published:** 2025-10-27

**Authors:** Mirah Lindsay, Susanna Huang

**Affiliations:** 1 Walton High School, Atlanta, Georgia, USA; 2 Georgia Institute of Technology, Atlanta, GA, USA

## Abstract

Crystallography has a wide range of applications in research, but this field is rarely discussed in primary and secondary school classrooms. While it is difficult to do cutting-edge, cancer-curing research in a high school laboratory, the intricacies of crystallography can still be introduced to students of any age. Through the Structural Nucleic Acid Anticancer Research Society (STARS), we have been able to help provide educational experiences and opportunities with crystal-growing and crystallography for students grades K-12th.

At the high school level, STARS has organized weekly club meetings to work with students in growing and experimenting with inorganic crystals. Students choose the best, most unique crystals of the ones they have grown to submit to the U.S. Crystal Growing Competition. Having this opportunity provides students with foundational knowledge of crystallography and can excite students about science, thus inspiring a new generation of scientists.

Beyond weekly meetings, high school STARS has also worked in conjunction with the collegiate STARS branch to offer crystallography workshops and camps. Through the workshops we provide students with the opportunity to experience wet lab research with lysozyme and gain a greater understanding of crystallography and its applications. Through the summer camp, which we hosted July 1-4, 2024, we allowed younger students to grow their own inorganic crystals and learn more about the science behind crystallography and its applications. Not only was it an educational experience for the students attending the camp, but it also a valuable opportunity for members of the high school STARS group to practice public speaking and science communication, both of which are valuable skills in a variety of professions.

STARS will continue to provide valuable lab experience through club meetings, workshops, camps, and more. We hope to continue to provide K-12th students with educational experiences that will help inspire the young scientists that are the future of this field of study.

